# Psychological resources for academic buoyancy: the roles of growth mindset and emotional intelligence in Chinese university students

**DOI:** 10.3389/fpsyg.2025.1580929

**Published:** 2025-06-05

**Authors:** Ran Liu

**Affiliations:** School of Marxism, Hohai University, Nanjing, China

**Keywords:** academic buoyancy, Chinese university students, emotional intelligence, growth mindset, mixed methods, resilience

## Abstract

**Background/introduction:**

While psychological resources like growth mindset (belief in malleable abilities) and trait emotional intelligence (EI; self-perceived emotional capabilities) are individually important in higher education, their dynamic interplay with resilience (capacity to recover from adversity) in contributing to academic buoyancy (students’ ability to navigate daily academic challenges) warrants further understanding, particularly within the demanding context of Chinese university undergraduates and the specific mediating mechanisms involved.

**Methods:**

This mixed-methods study employed a sequential explanatory design. The quantitative phase involved 381 undergraduates selected through stratified random sampling across several Chinese universities. Data were collected using established self-report instruments for growth mindset, trait EI, resilience, and academic buoyancy, and analyzed via structural equation modeling (SEM) to test a mediational model. The qualitative phase explored experiences of 20 purposefully selected students through reflective journals and a focus group, with data subjected to thematic analysis.

**Results:**

Quantitative findings revealed that both growth mindset and trait EI significantly predicted resilience, which, in turn, significantly and positively predicted academic buoyancy. Resilience fully mediated the pathways from both growth mindset and trait EI to academic buoyancy. Multi-group analysis indicated no statistically significant gender differences in these pathways. Qualitative data richly contextualized these findings, illustrating how students practically apply growth mindset and emotional regulation to navigate academic setbacks and highlighting the crucial role of social support.

**Discussion/conclusion:**

These findings underscore that interventions targeting growth mindset and EI may foster resilience to enhance academic buoyancy in higher education. The results highlight the importance of these psychological resources, especially within demanding academic contexts, and suggest that fostering resilience is a key mechanism for improving students’ ability to manage routine academic stressors.

## 1 Introduction

University life presents many academic demands, including difficult coursework, deadlines, low grades, and performance pressures (Law, 2007). These daily academic stressors, often perceived as minor compared to major life crises, accumulate and can impact student well-being and progress (Zhang and Zheng, 2017). In competitive educational systems, like those in many Chinese universities, these daily challenges can be pronounced, amplified by societal expectations for achievement and peer competition (Zhao, 2016). Managing these routine setbacks is crucial for academic engagement and success. While addressing major adversity is important, research also highlights academic buoyancy—the ability to “bounce back” from these common difficulties (Martin and Marsh, 2008). Indeed, recent studies consistently find that academic buoyancy predicts positive educational outcomes, including higher motivation, engagement, and academic performance (e.g., Bostwick et al., 2022; Guo et al., 2024; Liu et al., 2025; Putwain et al., 2022). However, the psychological mechanisms underpinning this specific capacity require closer examination, particularly regarding how key internal resources like growth mindset, emotional intelligence (EI), and resilience might work together (cf. Martin, 2013; Thomas et al., 2024). Elucidating this interplay, especially the mediating pathways, is vital not merely for general understanding but for designing more precise and effective interventions that target the specific mechanisms linking psychological resources to adaptive outcomes like buoyancy, thereby optimizing student support in demanding contexts like Chinese higher education.

Growth mindset—the belief that abilities develop through effort (Dweck, 2006; Yeager and Dweck, 2020)—is increasingly recognized as vital for positive academic outcomes (e.g., Claro and Loeb, 2024; Gazmuri, 2025). Students with a growth mindset view failures as learning opportunities and demonstrate greater persistence (Kim et al., 2022; Tang et al., 2019). Recent work further suggests growth mindset operates within broader systems including achievement goals, influencing outcomes like burnout (Altikulaç et al., 2024). Growth mindset interventions can improve grades and persistence (Macnamara and Burgoyne, 2023), and recent evaluations highlight their potential to foster positive responses to academic failure (Zhao et al., 2024) and enhance social–emotional outcomes (Jiang et al., 2024). Yet, the influence of growth mindset is nuanced, potentially moderated by contextual factors like socioeconomic status (King and Trinidad, 2021) and parental support (Chen et al., 2025; Zhao et al., 2024). Therefore, understanding how growth mindset interacts with other psychological resources and contextual variables is essential for leveraging its benefits across diverse student populations.

Emotional intelligence (EI), specifically trait EI defined as self-perceived emotional competencies (Wong and Law, 2002), also plays a key role in academic success (Gkintoni et al., 2025; MacCann et al., 2020). Trait EI, encompassing emotional appraisal and regulation, enables effective stress management and adaptive coping (Mohamed et al., 2025; Perera, 2016). These EI skills are foundational for resilience (Mohamed et al., 2025; Trigueros et al., 2020). Resilience—the ability to adapt and thrive amidst adversity (Masten, 2001; Smith et al., 2008)—is central to academic persistence and achievement (Derakhshan and Fathi, 2025; García-Martínez et al., 2022). Resilience is considered malleable (Nakhostin-Khayyat et al., 2024; Masten, 2001), potentially enhanced by growth mindset and EI through adaptive appraisals and emotional stability (Grover and Furnham, 2021; Mohamed et al., 2025). Given these established links, investigating how cognitive factors like growth mindset combine with emotional competencies (EI) and adaptive capacities (resilience) could reveal synergistic effects crucial for navigating the multifaceted demands of university life.

Despite evidence linking these constructs, key gaps persist that hinder the development of holistic student support strategies. First, academic buoyancy, while related, uniquely focuses on daily academic stressors, differing from resilience’s broader adversity focus (Martin, 2013). The combined influence of growth mindset, EI, and resilience on academic buoyancy specifically warrants further empirical exploration (cf. Putwain et al., 2022; Thomas et al., 2024). Second, resilience’s mediating role between growth mindset, EI, and buoyancy remains underexplored, particularly in non-Western contexts like Mainland China (Zeng et al., 2019), limiting culturally informed intervention design. Third, qualitative research explaining how these factors operate from the students’ perspective is scarce, limiting intervention development that resonates with lived experiences (Creswell and Plano Clark, 2017; Zhang, 2021). Addressing these gaps by examining the interplay and mechanisms within a specific cultural context is therefore a significant step toward creating more effective support systems.

To bridge these gaps, this mixed-methods study investigates the mediating role of resilience. Quantitatively, we use Structural Equation Modeling (SEM) to test if resilience mediates the effects of growth mindset and trait EI on academic buoyancy. Qualitatively, we explore the lived experiences of Chinese university students through reflective journals and a focus group to understand their coping mechanisms. This sequential explanatory design (Creswell and Plano Clark, 2017) provides both statistical validation and rich contextual insights.

Specifically, this study addresses: (1) growth mindset and trait EI as predictors of resilience and academic buoyancy; (2) resilience as a mediator; and (3) students’ descriptions of these constructs in managing daily academic challenges. Grounded in social-cognitive theory (Bandura, 1986, 1997) and positive psychology (Seligman, 2011), we hypothesize that resilience mediates the relationships, and qualitative data will reveal specific mechanisms. Ultimately, this research aims to develop a comprehensive framework illustrating how mindsets, emotions, and adaptive capacities enhance academic buoyancy, informing integrated interventions to foster student success and well-being in diverse educational settings.

## 2 Literature review and theoretical framework

### 2.1 Growth mindset: conceptualization and empirical evidence

Dweck (2006) influential work on growth mindset posits that individuals’ implicit beliefs about the malleability of intelligence and abilities significantly shape their academic behaviors and outcomes. This framework distinguishes between a fixed mindset, where intelligence is seen as static, leading to challenge avoidance and viewing effort as futile, and a growth mindset. The latter perceives intelligence as developable through effort, learning, and persistence, thereby encouraging individuals to embrace challenges and persevere (Burnette et al., 2023; Dweck, 2015; Yeager and Dweck, 2020). Consequently, educational initiatives often aim to foster growth mindsets by shifting students’ views of their abilities to enhance academic success (Han and Stieha, 2020; Zhang et al., 2025).

Substantial empirical evidence supports the benefits of a growth mindset. Research indicates students with a growth mindset generally achieve better academic results, demonstrate greater persistence, and exhibit more resilience (Bostwick et al., 2017; Fathi et al., 2024b; Gazmuri, 2025; Tang et al., 2019; Wang et al., 2020). For instance, growth mindset has been linked to increased commitment and grit, predicting long-term success (Tang et al., 2019), and positively influences achievement in challenging subjects like math and science (Bostwick et al., 2017; Claro and Loeb, 2024; Fathi et al., 2024a; Wang et al., 2020; Zhang et al., 2024). It is also associated with enhanced academic persistence, influencing college retention and completion (Kim et al., 2022; Li and Bates, 2020; McCabe et al., 2020), and promotes effective self-regulation and metacognitive strategies, as students view failure as a learning opportunity (Dong et al., 2023; Kim and Park, 2021; Macnamara and Burgoyne, 2023). Growth mindset interventions (GMIs) have proven effective in improving academic outcomes, especially for at-risk students (Broda et al., 2018; Corradi et al., 2019; Macnamara and Burgoyne, 2023). Recent studies confirm brief GMIs can shift mindsets and yield broader benefits, including improved social–emotional outcomes like reduced depression and aggression (Jiang et al., 2024), and better coping with academic failure, particularly in contexts like Chinese primary schools (Zhao et al., 2024). Such interventions may also buffer against achievement declines after setbacks (Zhao et al., 2024). However, the impact of growth mindset is not uniform. Nuances exist, such as differing outcomes based on growth mindset profiles and achievement goals (Altikulaç et al., 2024). Effectiveness can also be sensitive to contextual factors like socioeconomic status (King and Trinidad, 2021) and parental influences, including autonomy support and beliefs about failure (Chen et al., 2025; Zhao et al., 2024). Some research even questions its universal applicability, noting no significant link with scholastic aptitude in certain samples (Bahník and Vranka, 2017). These findings collectively highlight that while growth mindset significantly benefits academic outcomes by fostering a belief in malleable intelligence—thereby enhancing persistence, resilience, and performance—its effectiveness is often moderated by individual and contextual differences. Therefore, effective interventions require appropriate design, implementation, and contextualization, considering factors like parental support, especially for disadvantaged students (Broda et al., 2018; Corradi et al., 2019; Zhao et al., 2024).

### 2.2 The significance of emotional intelligence for academic outcomes

Emotional intelligence (EI)—the ability to effectively perceive, understand, manage, and utilize emotions—is a key psychological and educational construct due to its impact on academic performance, mental health, and resilience (Elfenbein and MacCann, 2017; Salovey and Mayer, 1990). Initially defined by Salovey and Mayer (1990) as processing and utilizing emotional information, EI evolved into various models, notably trait EI (Mayer and Salovey, 1997; Wong and Law, 2002). Trait EI, seen as developable, self-perceived emotional abilities (Mayer and Salovey, 1997; Mayer et al., 2008), is typically assessed via self-report (reflecting perceived emotional competence), distinguishing it from ability EI models that use performance tests (MacCann et al., 2020). Wong and Law (2002) identified four key trait EI dimensions: Self-Emotion Appraisal (SEA), Others’ Emotion Appraisal (OEA), Regulation of Emotion (ROE), and Use of Emotion (UOE). These, respectively, address self-awareness and decision-making (SEA); interpersonal effectiveness (OEA; Sánchez-Álvarez et al., 2020); emotional balance under stress (ROE; Perera, 2016; Trigueros et al., 2020); and using emotions to aid cognition and performance (UOE; Mayer and Salovey, 1997). Collectively, these dimensions shape how individuals process and respond to emotional information in academic and social contexts.

Extensive empirical research confirms EI’s critical role in fostering academic success and adaptive functioning. Numerous studies and meta-analyses show that higher EI, across both trait and ability conceptualizations, positively correlates with better academic performance in diverse educational settings (Gkintoni et al., 2025; MacCann et al., 2020; Mohamed Hashim et al., 2025; Perera and DiGiacomo, 2013). This link is associated with improved stress management, emotional regulation, social relationships (Peña-Sarrionandia et al., 2015; Sánchez-Álvarez et al., 2020; Zhoc et al., 2018), and, for students high in trait EI, more effective management of academic pressure and better learning strategies (Perera, 2016). For instance, strong Self-Emotion Appraisal (SEA) and Regulation of Emotion (ROE) skills predict reduced test anxiety and improved focus (Chew et al., 2013; Thomas and Zolkoski, 2020); proficient Others’ Emotion Appraisal (OEA) improves group work (Estrada et al., 2021); and effective Use of Emotion (UOE) enhances creative problem-solving (Halimi et al., 2021; Perera, 2016). Recent analyses further reveal higher EI positively influences deeper subject knowledge, active engagement, and real-world learning, beyond just grades (Mohamed Hashim et al., 2025). Moreover, EI significantly predicts resilience, aiding students in managing academic stress (Bunce et al., 2019; Mohamed et al., 2025; Thomas and Zolkoski, 2020; Trigueros et al., 2020). This is often achieved through enhanced emotional regulation, which mitigates stress and promotes recovery from setbacks (Grover and Furnham, 2021; Thomas and Zolkoski, 2020). Specific components like SEA and ROE are crucial here, linking to lower test anxiety (Trigueros et al., 2020), while interpersonal aspects like OEA enhance resilience via effective social support seeking and stronger teacher-student relationships (Chamizo-Nieto et al., 2021; Ononye et al., 2022). EI also buffers against academic burnout (Grover and Furnham, 2021) and positively impacts academic persistence and completion, with EI interventions potentially reducing dropout rates (Broda et al., 2018; Kim et al., 2022). While the EI-academic performance relationship is broadly positive, its strength can vary contextually, sometimes appearing more pronounced in demanding fields like healthcare (Okwuduba et al., 2021; Ononye et al., 2022) or showing nuances in how specific EI components contribute (Amponsah et al., 2024). Despite such variations, the compelling body of evidence strongly supports EI’s value, leading to consistent recommendations from recent scholarship for universities to integrate EI development into curricula and support strategies to improve overall student success and well-being (Amponsah et al., 2024; Gkintoni et al., 2025; Mohamed et al., 2025; Mohamed Hashim et al., 2025).

### 2.3 The role of resilience in student adaptation and success

Resilience, vital in education for understanding how students navigate academic adversity (Beltman and Mansfield, 2018; Masten, 2001; Windle, 2011), is conceptualized not as a fixed trait but as a dynamic process of adapting, recovering, and thriving despite significant challenges (Aburn et al., 2016; Masten, 2001). This capacity, which explains differential student success amidst obstacles, is commonly defined as the ability to recover from difficult situations and adjust effectively to adversity (Brewer et al., 2019; Masten, 2001; Smith et al., 2008). Contemporary perspectives emphasize its cultivatable nature (Masten, 2001), crucial for students needing to “bounce back” from academic setbacks (Milne et al., 2016; Smith et al., 2008); tools like the Brief Resilience Scale (BRS) measure this dynamic recoverability (Smith et al., 2008). Within education, academic resilience specifically denotes students’ ability to persevere, stay motivated, and succeed despite academic stressors like difficult coursework (Allan et al., 2014; García-Martínez et al., 2022; Li, 2017). It is distinct from general life resilience due to its focus on obstacles within the academic sphere (Bittmann, 2021; García-Martínez et al., 2022). Furthermore, modern views suggest resilience extends beyond individual characteristics, portraying it as a dynamic adaptive capacity developed through interactions within the broader educational ecosystem, emphasizing the interplay between individual attributes and supportive environmental contexts (Ross et al., 2024).

Empirical research consistently demonstrates academic resilience’s critical role in fostering student success and well-being. Resilient students are more likely to persist through adversity (Milne et al., 2016), effectively overcome setbacks, and sustain engagement, which contributes to improved academic performance, better retention, and enhanced overall well-being (Cassidy, 2015; Derakhshan and Fathi, 2025; Li, 2017). Resilience is pivotal for emotional regulation during academic challenges—essential for maintaining focus and motivation (MacPhee et al., 2015)—as resilient students typically exhibit better emotional control when responding to failures (Smith et al., 2008). It also underpins academic persistence, making students less likely to give up when facing difficulties (Edwards et al., 2016; Li, 2017; Montas et al., 2021) and, often alongside grit, predicts academic success (Montas et al., 2021). For instance, Chinese students with higher resilience cope more effectively with exam pressure, achieving better results (Li, 2017). Recent studies on its psychological mechanisms highlight the significant interplay of cognitive and self-regulatory functions; self-regulation, for example, positively predicts resilience, a relationship partially mediated by cognitive flexibility—the ability to adapt thinking and behavior (Nakhostin-Khayyat et al., 2024). Thus, fostering foundational skills like self-regulation (also a key EI component linked to academic success; Herut et al., 2024) appears crucial for building resilience. Beyond internal factors, external elements like social support from peers, family, and instructors also significantly foster resilience (Allan et al., 2014), and positive self-concept mediates the link between resilience and achievement (García-Martínez et al., 2022). Moreover, resilience frequently acts as a crucial mediator between other psychological traits, like emotional intelligence, and academic outcomes such as reduced test anxiety or effective management of university transitions (Bittmann, 2021; Trigueros et al., 2020).

### 2.4 Academic buoyancy: definition, distinction, and outcomes

Academic buoyancy, a student’s capacity to navigate and rebound from common daily academic challenges like poor grades or deadline pressures, is increasingly recognized in educational psychology for fostering student success, motivation, and well-being (Martin, 2013; Martin and Marsh, 2008). Crucially, it differs from academic resilience: buoyancy addresses routine daily stressors, making it highly relevant for most students, whereas resilience concerns responses to significant adversity (Datu and Yang, 2021; Martin, 2013; Putwain et al., 2020). This distinction highlights the different coping mechanisms involved. Martin and Marsh (2008) defined academic buoyancy as the capacity to “bounce back” from such routine setbacks, differentiating it from broader resilience models (Putwain et al., 2023). Effective management of these frequent daily pressures is vital for students’ sustained academic engagement and consistent performance (Collie et al., 2015; Putwain et al., 2022). Buoyant students typically adeptly manage these setbacks, recover from temporary performance dips, and maintain a positive learning approach (Miller et al., 2013; Mohammad Hosseini et al., 2024).

Empirical research consistently links academic buoyancy to a range of positive educational outcomes. Buoyant students exhibit greater engagement and motivation when tackling academic challenges (Bostwick et al., 2022; Liu et al., 2025; Thomas and Allen, 2021), and higher buoyancy in high school predicts sustained achievement, even amidst adversity (Collie et al., 2015). This capacity also fosters greater agency and self-efficacy, as buoyant students often perceive more control over their academic trajectories (Collie et al., 2017). Furthermore, academic buoyancy is associated with more positive achievement-related emotions and fewer negative emotional responses to challenges, which supports higher performance (Putwain et al., 2022). This positive link with motivation and engagement has been observed across diverse cultural contexts, including among Filipino students (Datu and Yang, 2021). Beyond academic performance, buoyancy significantly relates to student well-being; for instance, more buoyant primary school students demonstrate higher overall well-being, better emotional regulation, and reduced stress (Miller et al., 2013).

Recent studies also illuminate academic buoyancy’s function as a key psychological mechanism. For instance, among Chinese doctoral students, it significantly mediates the pathway from academic self-concept to tangible academic performance (Guo et al., 2024). Buoyancy also plays a protective role against negative experiences like learning burnout, particularly for EFL learners in China, where it mediates the mitigating effects of social support from teachers and peers (Fu, 2024). Key psychological predictors of academic buoyancy include growth mindset, which encourages viewing challenges as learning opportunities (Martin, 2013; Thomas et al., 2024; Valdez, 2024), and emotional intelligence (EI), which equips students to manage academic emotional demands (Thomas and Allen, 2021). However, the interplay of these predictors can be complex. Recent research on EI, self-compassion, and achievement goals, for example, revealed intricate serial mediation effects where high self-compassion (though boosted by EI) sometimes negatively linked with certain achievement goals, thereby indirectly decreasing buoyancy (Thomas et al., 2024). Resilience, too, is consistently associated with academic buoyancy (Weißenfels et al., 2023; Zhang, 2021), and factors like teacher support and academic self-efficacy further contribute to this interconnected web (Weißenfels et al., 2023; Zhang, 2021). Collectively, these studies underscore academic buoyancy as a critical mediator and outcome, influenced by self-perceptions, social context, and a complex network of emotional and motivational orientations (Fu, 2024; Guo et al., 2024; Thomas et al., 2024). Ultimately, fostering academic buoyancy is crucial for helping students effectively manage everyday academic challenges and thrive.

### 2.5 Theoretical rationale, model development, and hypotheses

This study draws on social-cognitive theory (Bandura, 1986, 1997) and positive psychology (Masten, 2018; Seligman, 2011), which highlight the role of personal beliefs, self-regulation, and psychological strengths in navigating challenges and fostering well-being. We investigate the interconnected roles of growth mindset, trait EI, and resilience in fostering university students’ academic buoyancy—their capacity to manage and recover from everyday academic setbacks (Martin and Marsh, 2008). While these constructs are individually linked to academic outcomes, their dynamic interplay within a comprehensive mediational model—particularly in demanding non-Western academic contexts like Mainland China—remains underexplored.

**Figure 1:**
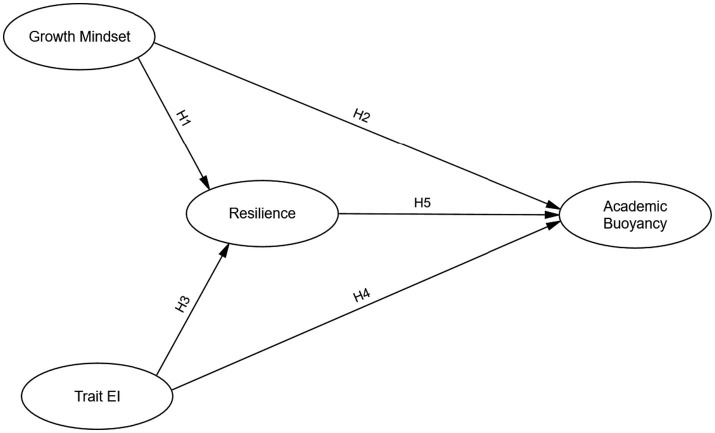
Hypothesized structural model of the relationships between growth mindset, trait emotional intelligence, resilience, and academic buoyancy, including direct and mediated pathways.

The current study proposes and tests a specific mediational model, visually presented in Figure 1, where growth mindset and trait EI are conceptualized as foundational psychological resources contributing to resilience. This enhanced resilience, in turn, is posited as a key factor in fostering students’ academic buoyancy. Social-cognitive theory (Bandura, 1986) suggests that self-efficacy beliefs—bolstered by a growth mindset (the belief in developable intelligence; Dweck, 2006; Yeager and Dweck, 2020) and effective emotional self-regulation (a component of EI; Bandura, 1997; Schunk and DiBenedetto, 2020)—are crucial for perseverance and adaptive coping. Students who believe their abilities can grow and who can effectively manage their emotions are likely to be more resilient. Academic buoyancy can thus be viewed as a manifestation of this resilience within the daily academic sphere (Collie et al., 2015; Martin, 2013). Positive psychology further supports this integrated perspective by highlighting how such psychological strengths can lead to thriving amidst routine academic stressors (Masten, 2018; Seligman, 2011; Ungar, 2011).

Building on this foundation and the model depicted in Figure 1, we derive specific hypotheses. First, regarding growth mindset, its emphasis on development through effort (Dweck, 2006) is expected to directly enhance persistence and adaptation, thereby fostering resilience (Bostwick et al., 2017; Tang et al., 2019; Zeng et al., 2019). The learning strategies and persistence it promotes (Macnamara and Burgoyne, 2023) should also directly aid in managing routine academic stressors, positively influencing academic buoyancy (Martin, 2013; Valdez, 2024). Therefore, we hypothesized:

*H1*: *Growth mindset will positively predict resilience among university students.*

*H2*: *Growth mindset will positively predict academic buoyancy among university students.*

Second, concerning trait emotional intelligence, its role in understanding and managing emotions (Wong and Law, 2002) is critical for navigating academic life. Higher trait EI facilitates stress regulation and motivation (Perera, 2016), which are fundamental to developing resilience (Trigueros et al., 2020; Grover and Furnham, 2021). Such emotional management should also directly equip students to handle daily academic challenges, contributing to academic buoyancy (Thomas and Allen, 2021; Thomas et al., 2024). Consequently, we hypothesized:

*H3: Trait emotional intelligence will positively predict resilience among university students.*

*H4: Trait emotional intelligence will positively predict academic buoyancy among university students.*

Third, as illustrated in our model (Figure 1), we expect a direct positive relationship between resilience and academic buoyancy. Given that resilience involves adapting to stress (Masten, 2001) and academic buoyancy is a domain-specific application of this adaptive capacity to routine academic setbacks (Martin and Marsh, 2008; Martin, 2013), higher general resilience should logically lead to greater academic buoyancy (Zhang, 2021). Accordingly, we hypothesized:

*H5*: *Resilience will positively predict academic buoyancy among university students.*

Finally, a central tenet of our proposed model (Figure 1) is the mediating role of resilience. We posit that the positive effects of growth mindset and trait EI on academic buoyancy are channeled, at least in part, through their impact on building resilience. This pathway aligns with social-cognitive perspectives where adaptive beliefs and skills (growth mindset, EI) enhance broader coping capacities (resilience), which then facilitate specific positive outcomes (academic buoyancy) (Bandura, 1997). Prior research supports growth mindset and EI as predictors of resilience (MacCann et al., 2020; Trigueros et al., 2020; Zeng et al., 2019) and resilience as a mediator for academic outcomes (Bittmann, 2021; Valdez, 2024; Putwain et al., 2022). Based on this, we further hypothesized:

*H6*: *Resilience will mediate the positive relationship between growth mindset and academic buoyancy.*

*H7*: *Resilience will mediate the positive relationship between trait emotional intelligence and academic buoyancy.*

These seven hypotheses (see Figure 1 for the full structural model) guide the quantitative phase of this study. While research on these constructs individually is growing, integrated mixed-methods studies examining these specific mediational pathways in the Chinese higher education context remain limited (Macnamara and Burgoyne, 2023). To address this gap and gain a holistic understanding, we employed a sequential explanatory mixed-methods design (Creswell and Plano Clark, 2017), combining statistical model testing with rich qualitative data for comprehensive exploration. The quantitative phase utilized SEM to test the hypothesized direct and indirect effects. Subsequently, the qualitative phase, through reflective journals and a focus group, aimed to explain and enrich these findings by exploring students’ lived experiences of how these psychological resources operate in their academic lives (Creswell and Plano Clark, 2017; Zhang, 2021). This triangulation approach (Denzin and Lincoln, 2018) helps overcome single-method limitations.

## 3 Methods

### 3.1 Participants and procedures

An initial total of 411 university students from 10 major universities across five provinces in Mainland China were recruited and began participation in the quantitative phase of this study. Participants were recruited using a stratified random sampling approach to ensure adequate representation across different geographical regions, academic disciplines, and year levels, capturing the diversity of the Chinese higher education system and enhancing the external validity of the findings. The final quantitative sample comprised 235 female students (57.2%) and 176 male students (42.8%), with ages ranging from 18 to 25 years (*M* = 20.7, SD = 2.3). The sample included students from various academic disciplines: humanities (35%), social sciences (30%), natural sciences (20%), and engineering (15%), reflecting a broad range of educational backgrounds. Furthermore, to enhance the generalizability of the results, students from all undergraduate years were included: 27% first year, 25% second year, 28% third year, and 20% final year of study. All participants were full-time undergraduate students, reporting an average Grade Point Average (GPA) of 3.5 (on a 4.0 scale), indicating a relatively high-performing cohort.

Quantitative data collection was conducted using online questionnaires administered via Qualtrics, a secure and widely used platform. Prior to accessing the questionnaire, participants received detailed instructions and were allotted approximately 20 min for completion. To ensure data quality and minimize careless responses, attention-check questions were randomly embedded within the survey. Participants who failed these checks or completed the survey in an unreasonably short time were excluded from analysis. This initial data screening process led to the removal of 25 participants. This resulted in an intermediate sample of 386 participants whose data were carried forward for preliminary analysis. The demographic characteristics reported subsequently pertain to this sample of 386 students.

This sample of 386 students comprised 221 female students (57.2%) and 165 male students (42.8%), with ages ranging from 18 to 25 years (*M* = 20.7, *SD* = 2.3). The sample included students from various academic disciplines: humanities (35%), social sciences (30%), natural sciences (20%), and engineering (15%), reflecting a broad range of educational backgrounds. Furthermore, to enhance the generalizability of the results, students from all undergraduate years were included: 27% first year, 25% second year, 28% third year, and 20% final year of study. All participants were full-time undergraduate students, reporting an average Grade Point Average (GPA) of 3.5 (on a 4.0 scale), indicating a relatively high-performing cohort.

Participation was voluntary, and all students provided written informed consent before data collection. Ethical standards were rigorously maintained, with the study protocol, including recruitment and data handling, reviewed and approved by the Institutional Review Board (IRB) of the Hohai University. Participants were assured of data confidentiality and informed of their right to withdraw at any time without penalty. Small academic credits or gift vouchers were offered as incentives to encourage participation and support the required sample size for statistical power.

Following the quantitative phase, qualitative data were collected in two stages. The first stage involved reflective journals submitted through WeChat over 4 weeks. WeChat was chosen for its widespread use among Chinese students, facilitating convenient and regular submissions. Participants received weekly prompts to guide their reflections on specific experiences, ensuring comprehensive data collection. The second qualitative stage consisted of a focus group discussion conducted virtually via Zoom. Zoom was utilized for its familiarity and flexibility, accommodating diverse student schedules while maintaining an interactive environment. All participants provided informed consent for audio recording of the focus group session.

### 3.2 Instrumentation and data collection

To assess the study’s key constructs, a range of established scales were utilized alongside qualitative data collection methods. First, the Growth Mindset Scale (Dweck, 2006) evaluated students’ beliefs about the malleability of intelligence and academic abilities. This self-report instrument featured items capturing growth mindset beliefs (e.g., “You can always substantially change how intelligent you are”), with responses on a 5-point Likert scale (1 = Strongly Disagree to 5 = Strongly Agree). For cultural appropriateness in the Chinese context, the scale underwent rigorous forward- and back-translation by bilingual experts, followed by a successful pilot test with 100 university students. The Chinese version demonstrated strong internal consistency (Cronbach’s *α* = 0.82 for the growth mindset subscale). Factor analysis supported its unidimensionality, and confirmatory factor analysis (CFA) affirmed acceptable construct validity (χ^2^/df = 2.01, CFI = 0.95, TLI = 0.93, RMSEA = 0.05 [0.04–0.06], RMSR = 0.04).

Next, trait emotional intelligence (EI) was measured using a Chinese version of the Wong and Law Emotional Intelligence Scale (WLEIS; Wong and Law, 2002). This 16-item self-report scale assessed four key EI dimensions based on Mayer and Salovey’s (1997) framework: Self-Emotion Appraisal (SEA), Others’ Emotion Appraisal (OEA), Regulation of Emotion (ROE), and Use of Emotion (UOE), each with four items (e.g., for SEA: “I always know whether or not I am happy”; for OEA: “I am sensitive to the feelings and emotions of others”; for UOE: “I always tell myself I am a competent person”; for ROE: “I am quite capable of controlling my own emotions”). Participants responded on a 5-point Likert scale (1 = *Strongly Disagree* to 5 = *Strongly Agree*). The WLEIS has shown robust psychometrics previously; in this study, Cronbach’s α for the dimensions ranged from 0.80 to 0.87, indicating high reliability. A post-translation CFA further supported strong construct validity (χ^2^/ *df* = 2.23, CFI = 0.96, TLI = 0.94, RMSEA = 0.05 [0.04–0.07], RMSR = 0.04).

Participants’ ability to recover from adversity was measured using the Brief Resilience Scale (BRS; Smith et al., 2008). The BRS is a six-item scale with items directly reflecting resilience (e.g., “I tend to bounce back quickly after hard times”) and reverse-scored items. Responses were recorded on a 5-point Likert scale (1 = *Strongly Disagree* to 5 = *Strongly Agree*), with summed scores indicating resilience level. The BRS, known for strong psychometrics, showed high internal consistency in this study (Cronbach’s α = 0.84). CFA confirmed its suitability (χ^2^/*df* = 2.75, CFI = 0.92, TLI = 0.90, RMSEA = 0.06 [0.04–0.08], RMSR = 0.05).

Finally, academic buoyancy—students’ ability to navigate everyday academic challenges—was assessed with the Academic Buoyancy Scale (ABS; Martin and Marsh, 2008). This concise, four-item scale measures capacity to overcome academic stress and setbacks (e.g., “I can deal with setbacks in my academic work”), using a 5-point Likert scale (1 = *Strongly Disagree* to 5 = *Strongly Agree*). Higher scores indicate greater academic buoyancy. The scale demonstrated strong reliability in this study (Cronbach’s α = 0.81) and acceptable construct validity via CFA (χ^2^/*df* = 1.98, CFI = 0.95, TLI = 0.93, RMSEA = 0.05 [0.03–0.07], RMSR = 0.03), establishing it as a reliable measure of academic buoyancy for this student population.

Following quantitative data collection, qualitative data were gathered via reflective journals. A subset of 20 participants, selected based on resilience scale scores to ensure diverse experiences, contributed weekly journals via WeChat for 4 weeks. These journals, approximately 500–800 words each and guided by prompts, aimed to provide deeper insights into personal experiences with academic buoyancy, resilience, growth mindset, and EI in their academic lives. Prompts encouraged exploration of navigating setbacks, emotional regulation, applying growth mindset principles to overcome challenges, and reflecting on incidents testing EI and resilience. Clear guidelines emphasized specific examples of academic challenges, emotions, and coping strategies, fostering honest self-reflection. These journals offered rich qualitative data and facilitated real-world engagement with the study’s constructs.

Complementing the journals, a 90-min focus group was conducted with the same 20 students after their journal submissions. This session aimed to expand on journal themes and foster collective discussion about shared experiences related to academic buoyancy, growth mindset, emotional intelligence, and resilience. A trained facilitator moderated the Zoom-based session, ensuring a neutral and open environment conducive to participant sharing. Participants were encouraged to discuss their journal writing and elaborate on strategies for overcoming academic challenges. The moderator used semi-structured questions, building on journal themes, to guide the discussion, exploring topics such as students’ conceptualizations of growth mindset and manifestations of EI in academic interactions. The audio-recorded and verbatim transcribed session provided a dynamic, interactive context for participants to co-construct meanings around their experiences, thereby enriching the quantitative findings.

Given that all data were collected via self-report questionnaires at a single time point, common method variance (CMV) was assessed using Harman’s single-factor test (Podsakoff et al., 2003). An exploratory factor analysis (EFA) was conducted on all items from the four main scales (Growth Mindset, WLEIS, BRS, Academic Buoyancy Scale). The results of the unrotated EFA indicated the presence of multiple factors with eigenvalues greater than 1.0, and the first extracted factor accounted for 36.8% of the total variance. As this value is below the commonly accepted threshold of 50%, it suggests that CMV was not a substantial concern in this study.

Prior to SEM, preliminary analyses were conducted on the intermediate dataset of 386 participants to ensure data assumptions were met. Normality, assessed via skewness and kurtosis (values within ±2), was confirmed. Multicollinearity was ruled out with Variance Inflation Factor (VIF) values below 2.0 for all predictor variables in the regression models used within SEM. Multivariate outlier analysis using Mahalanobis distance led to the removal of five additional cases. Combined with the initial removal of 25 participants due to attention-check failures and rapid response times (as described in Participants and Procedures), the final sample for SEM comprised 381 participants, deemed suitable for the analysis.

### 3.3 Data analysis

For the quantitative phase, covariance-based structural equation modeling (CB-SEM) using AMOS 26.0 was employed to evaluate the hypothesized relationships between growth mindset, trait emotional intelligence (EI), resilience, and academic buoyancy. This approach was selected primarily because the study’s main objective was theory testing—specifically, confirming a pre-specified mediational model derived from established theoretical frameworks and prior empirical evidence (e.g., Hair et al., 2022; Kline, 2023). CB-SEM is well-suited for such confirmatory purposes, emphasizing model-data fit via global indices (Hu and Bentler, 1999). Furthermore, the utilization of established scales with reflective indicators in this study, alongside an adequate sample size for achieving stable parameter estimates (Rigdon, 2016), aligned with the strengths of CB-SEM in rigorously testing the hypothesized direct and indirect effects, making it more suitable than alternatives like PLS-SEM for our research goals. To determine the goodness of fit for the model, standard fit indices were applied, including the Tucker-Lewis Index (TLI), Comparative Fit Index (CFI), Root Mean Square Error of Approximation (RMSEA), and the Standardized Root Mean Square Residual (SRMR), following the guidelines established by Hu and Bentler (1999). Acceptable model fit was defined as TLI and CFI values exceeding 0.90, RMSEA values below 0.08, and SRMR values below 0.08. In addition, the indirect effects of growth mindset and emotional intelligence on academic buoyancy, through the mediating role of resilience, were examined using the bootstrap method with 5,000 resamples to generate bias-corrected confidence intervals (Preacher and Hayes, 2008).

For the qualitative data, the reflective journals and focus group transcripts were analyzed using thematic analysis (Braun and Clarke, 2006), which involved several iterative steps to ensure a rigorous and thorough understanding of the data. First, both data sets were read and re-read by the primary researcher to develop a deep familiarity with the content. Following transcription, the data were imported into NVivo (Version 14) qualitative data analysis software to facilitate systematic management and coding. Initial coding was conducted within NVivo, whereby segments of the transcripts relevant to recurring ideas and patterns were identified and assigned initial codes, closely aligned with the study’s research questions regarding experiences with growth mindset, emotional intelligence, resilience, and academic buoyancy. This process involved careful line-by-line coding initially, followed by a review phase to consolidate and refine the preliminary codes. Codes were then iteratively reviewed, compared, and grouped into broader potential themes within the software, allowing for organization and exploration of relationships between codes. These potential themes were then further refined and finalized based on their prevalence and relevance across both the journal and focus group data sets, ensuring they accurately captured participants’ experiences with growth mindset, emotional intelligence, resilience, and academic buoyancy.

To ensure credibility and trustworthiness of the qualitative findings, triangulation of the journal and focus group data was employed, allowing for the corroboration of themes across different data sources (Patton, 1999). The data were also subjected to inter-coder reliability checks, where a second researcher independently reviewed and coded a subset of the transcripts (approximately 20%). An agreement rate of 85% was achieved between the coders, suggesting a high level of consistency and reliability in the coding process. Discrepancies were resolved through discussion and consensus, ensuring that all themes were well-supported by the data. This combination of thematic analysis supported by NVivo, triangulation, and inter-coder reliability contributed to the robustness of the qualitative analysis, allowing for a richer interpretation of participants’ lived experiences.

## 4 Findings

### 4.1 Quantitative results

#### 4.1.1 Descriptive statistics and correlation analysis

Table 1 presents the descriptive statistics for growth mindset, trait emotional intelligence (*EI*), resilience, and academic buoyancy. Mean scores indicated relatively high levels across all constructs. Specifically, growth mindset (*M* = 4.12, *SD* = 0.62) and trait *EI* (*M* = 4.01, *SD* = 0.57) means suggest participants generally believe in developing abilities and possess strong emotional skills. Academic buoyancy also showed a high mean (*M* = 3.95, *SD* = 0.65), indicating perceived competence in managing academic setbacks. Resilience, the hypothesized mediator, exhibited a moderate mean (*M* = 3.85, *SD* = 0.60), suggesting a capacity for recovery from academic challenges.

**Table 1:** Descriptive statistics and inter-correlations.

Variable	*M*	SD	1	2	3	4
1. Growth Mindset	4.12	0.62	1			
2. Trait EI	4.01	0.57	0.43***	1		
3. Resilience	3.85	0.60	0.43***	0.47***	1	
4. Academic buoyancy	3.95	0.65	0.39***	0.42***	0.55***	1

***p < 0.001.

To assess the relationships between variables, correlation analyses were conducted, controlling for GPA and year of study. Even when accounting for these factors, growth mindset remained significantly correlated with trait *EI* (*r* = 0.36, *p* < 0.001) and academic buoyancy (*r* = 0.33, *p* < 0.001). Notably, resilience showed the strongest correlation with academic buoyancy (*r* = 0.50, *p* < 0.001), emphasizing its central role in navigating academic stressors.

The correlation matrix (Table 1) further detailed variable interrelations, revealing significant positive correlations in expected directions. Growth mindset was positively correlated with resilience (*r* = 0.43, *p* < 0.001) and academic buoyancy (*r* = 0.39, *p* < 0.001), suggesting that a stronger growth mindset is associated with greater resilience and academic buoyancy. Similarly, trait *EI* showed positive correlations with resilience (*r* = 0.47, *p* < 0.001) and academic buoyancy (*r* = 0.42, *p* < 0.001), indicating emotional intelligence is linked to these adaptive traits. The particularly strong correlation between resilience and academic buoyancy (*r* = 0.55, *p* < 0.001) supports resilience’s hypothesized mediating role.

#### 4.1.2 Confirmatory factor analysis of the measurement model

Confirmatory factor analysis (CFA) assessed the measurement model for growth mindset, trait emotional intelligence, resilience, and academic buoyancy. The model demonstrated excellent fit: χ^2^ (146) = 215.72, *p* < 0.001, CFI = 0.96, TLI = 0.94, RMSEA = 0.04, SRMR = 0.05. These indices indicate a strong model fit, with RMSEA and SRMR well below 0.08, and CFI and TLI exceeding the 0.90 threshold for acceptable fit (Hu and Bentler, 1999).

All factor loadings for observed variables were statistically significant (*p* < 0.001) and exceeded 0.50, indicating reliable measurement of latent constructs. For instance, growth mindset items loaded from 0.61 to 0.74, emotional intelligence items from 0.65 to 0.78, resilience items from 0.68 to 0.81, and academic buoyancy items from 0.66 to 0.82. These substantial loadings, detailed in Table 2, provide strong evidence for the construct validity of the measurement model.

**Table 2:** Factor loadings for latent constructs.

Latent construct	Item	Factor loading	*p*-value
Growth mindset	Item 1	0.74	<0.001
Growth mindset	Item 2	0.68	<0.001
Growth mindset	Item 3	0.61	<0.001
Growth mindset	Item 4	0.72	<0.001
Growth mindset	Item 5	0.65	<0.001
Growth mindset	Item 6	0.70	<0.001
Growth mindset	Item 7	0.63	<0.001
Growth mindset	Item 8	0.75	<0.001
Growth mindset	Item 9	0.67	<0.001
Growth mindset	Item 10	0.71	<0.001
Trait emotional intelligence	Item 1	0.78	<0.001
Trait emotional intelligence	Item 2	0.72	<0.001
Trait emotional intelligence	Item 3	0.65	<0.001
Trait emotional intelligence	Item 4	0.70	<0.001
Trait emotional intelligence	Item 5	0.71	<0.001
Trait emotional intelligence	Item 6	0.69	<0.001
Trait emotional intelligence	Item 7	0.75	<0.001
Trait emotional intelligence	Item 8	0.68	<0.001
Trait emotional intelligence	Item 9	0.73	<0.001
Trait emotional intelligence	Item 10	0.66	<0.001
Trait emotional intelligence	Item 11	0.77	<0.001
Trait emotional intelligence	Item 12	0.70	<0.001
Trait emotional intelligence	Item 13	0.67	<0.001
Trait emotional intelligence	Item 14	0.74	<0.001
Trait emotional intelligence	Item 15	0.69	<0.001
Trait emotional intelligence	Item 16	0.72	<0.001
Resilience	Item 1	0.81	<0.001
Resilience	Item 2	0.75	<0.001
Resilience	Item 3	0.68	<0.001
Resilience	Item 4	0.72	<0.001
Resilience	Item 5	0.65	<0.001
Resilience	Item 6	0.70	<0.001
Resilience	Item 7	0.73	<0.001
Resilience	Item 8	0.76	<0.001
Resilience	Item 9	0.69	<0.001
Resilience	Item 10	0.71	<0.001
Resilience	Item 11	0.79	<0.001
Resilience	Item 12	0.74	<0.001
Academic buoyancy	Item 1	0.82	<0.001
Academic buoyancy	Item 2	0.71	<0.001
Academic buoyancy	Item 3	0.66	<0.001
Academic buoyancy	Item 4	0.75	<0.001
Academic buoyancy	Item 5	0.72	<0.001
Academic buoyancy	Item 6	0.70	<0.001
Academic buoyancy	Item 7	0.68	<0.001
Academic buoyancy	Item 8	0.76	<0.001

#### 4.1.3 Structural equation model results: direct and indirect effects

To investigate the relationships among growth mindset, trait emotional intelligence (EI), resilience, and academic buoyancy, SEM was employed using AMOS 26.0. The model tested both the direct effects of growth mindset and emotional intelligence on academic buoyancy and the indirect effects of these variables through resilience.

**Figure 2:**
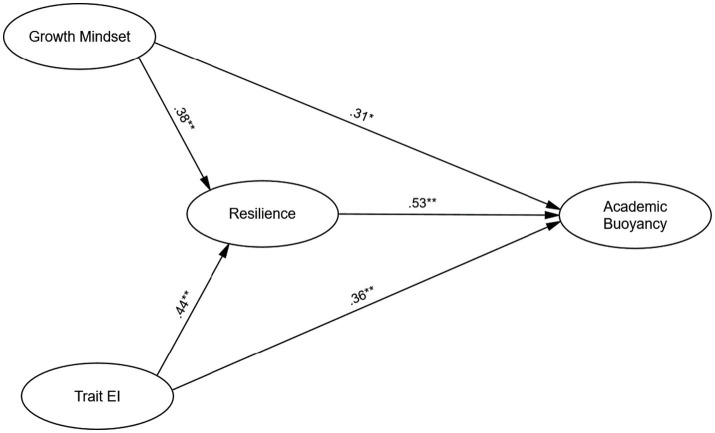
The model of academic buoyancy based on trait emotional intelligence, growth mindset, and resilience.

The structural model exhibited good fit to the data: χ^2^(148) = 222.18, *p* < 0.001, CFI = 0.95, TLI = 0.93, RMSEA = 0.05, and SRMR = 0.04. As indicated in Figure 2, growth mindset was found to have a significant positive direct effect on resilience (*β* = 0.38, *p* = 0.002) and a significant direct effect on academic buoyancy (*β* = 0.31, *p* = 0.034). Similarly, trait emotional intelligence had a significant direct effect on resilience (*β* = 0.44, *p* < 0.001) and a significant direct effect on academic buoyancy (*β* = 0.36, *p* = 0.007). The relationship between resilience and academic buoyancy was (Table 3).

**Table 3:** Standardized direct effects with standard errors.

Predictor	Outcome	β	*SE*	*p*-value
Growth mindset	Resilience	0.38	0.05	<0.001
Trait emotional intelligence	Resilience	0.44	0.04	<0.001
Resilience	Academic buoyancy	0.53	0.03	<0.001
Growth mindset	Academic buoyancy	0.31	0.06	<0.025
Trait emotional intelligence	Academic buoyancy	0.36	0.04	<0.007

The indirect effects of both growth mindset and emotional intelligence on academic buoyancy through resilience were significant. Specifically, the indirect effect of growth mindset on academic buoyancy via resilience was *β* = 0.21, *p* = 0.004, while the indirect effect of emotional intelligence on academic buoyancy through resilience was *β* = 0.24, *p* < 0.001. These findings underscore the critical mediating role of resilience in the relationship between growth mindset, emotional intelligence, and academic buoyancy. The total effects (direct + indirect) of growth mindset and emotional intelligence on academic buoyancy are presented in Table 4.

**Table 4:** Standardized indirect and total effects.

Predictor	Outcome	Indirect effect	*p*-value	Total effect	*p*-value
Growth mindset	Academic buoyancy	0.21	<0.004	0.52	<0.001
Trait emotional intelligence	Academic buoyancy	0.24	<0.001	0.60	<0.001

The significance of the mediation effects was further confirmed using the bootstrap method with 5,000 resamples to calculate bias-corrected confidence intervals (CIs). Resilience significantly mediated the relationship between growth mindset and academic buoyancy (95% CI [0.12, 0.32]) and between emotional intelligence and academic buoyancy (95% CI [0.15, 0.36]). As the confidence intervals did not include zero, the mediation effects were statistically significant, further confirming resilience’s pivotal role in promoting academic buoyancy.

#### 4.1.4 Multi-group SEM for gender differences

A multi-group SEM analysis was conducted to investigate potential gender differences in the structural relationships. The model fit indices for both male and female groups indicated acceptable fit: for males [χ^2^(148) = 219.34, CFI = 0.94, RMSEA = 0.05], and for females [χ^2^(148) = 226.15, CFI = 0.93, RMSEA = 0.05]. A chi-square difference test showed no significant differences in path coefficients between males and females (Δχ^2^ = 5.21, *p* = 0.11), suggesting that the relationships among growth mindset, emotional intelligence, resilience, and academic buoyancy were consistent across genders.

### 4.2 Qualitative findings: thematic analysis of student experiences

The qualitative phase explored the interplay of growth mindset, trait EI, and resilience in academic buoyancy. This was achieved through thematic analysis (Braun and Clarke, 2006) of reflective journals and a focus group, aiming to provide rich context for the quantitative findings. The analysis, supported by NVivo (Version 14) software, incorporated rigorous procedures such as data triangulation and inter-coder reliability checks to ensure the trustworthiness of the findings (further details are provided in the Data Analysis section). This process revealed key themes illuminating the practical application of these constructs in students’ academic lives: navigating academic challenges, emotional regulation strategies, growth mindset in action, resilience in bouncing back, and social support as a buffer.

A central theme was students’ experiences of navigating academic challenges, including exam failures, difficult coursework, and academic pressure, which they described as tests of intellectual and emotional endurance requiring resilience. For instance, one participant, after failing a math mid-term and initially feeling “the world was crashing down,” demonstrated a growth orientation by stating, “instead, I reviewed where I went wrong and made a study plan. Every failure was just another chance to improve” (Participant 8, Reflective Journal). Another student described managing deadline pressure by drawing on past coping experiences: “There were times when I had multiple deadlines… But I had to remind myself that I’ve faced challenges like this before and come out stronger each time” (Participant 15, Focus Group). These accounts illustrate how reframing setbacks as learning opportunities reflected a growth mindset, while resilience enabled perseverance, and effective emotional navigation suggested the role of emotional intelligence in overcoming obstacles.

Participants frequently highlighted the use of emotional regulation strategies as crucial for managing stress and maintaining buoyancy, particularly those with higher reported trait EI. One student exemplified cognitive reappraisal by “step[ping] back and think[ing]… One bad grade does not mean I’m a bad student. I needed to calm down” (Participant 3, Focus Group), indicating deliberate emotional management. Another described using both intra- and inter-personal strategies: “When I feel overwhelmed, I make sure to acknowledge my emotions… Journaling helps… talking to my friends gives me perspective” (Participant 12, Reflective Journal). Such approaches align with EI’s self-emotion appraisal and regulation dimensions, and the ability to pause, reflect, and manage emotions during academic stress exemplified EI’s contribution to their resilience, consistent with quantitative results.

The qualitative data also provided clear examples of students actively applying growth mindset principles. They often viewed challenges as learning opportunities, emphasizing that intelligence is malleable and can evolve. One student, after initial discouragement with chemistry, realized “this was part of learning,” then “reached out to my professor, asked for feedback, and studied even harder… I saw gradual improvement, and that kept me motivated” (Participant 5, Reflective Journal). This demonstrated adaptive behaviors linked to a growth mindset. Similarly, another student confronted a fixed mindset about physics: “Initially, I thought I wasn’t cut out for physics. But I reminded myself that intelligence is not fixed… I doubled down on practicing problems… Now, I’m much more confident” (Participant 9, Focus Group). These narratives underscore how a belief in flexible intelligence empowered persistence, mirroring quantitative findings where growth mindset predicted resilience.

Resilience and academic recovery, or bouncing back from setbacks, was another prominent theme. Students described resilience as persevering, adapting, and recovering from failure, often perceiving setbacks as temporary. One participant detailed overcoming a significant academic failure: “I failed my final exam in statistics… It hit me hard… But after some reflection, I decided to retake the exam. I studied more effectively this time, and when I passed, it felt like a huge personal victory” (Participant 18, Reflective Journal). Another emphasized resilience’s cumulative nature: “Every time I face a challenge and bounce back, it builds my confidence… It’s almost like a muscle” (Participant 7, Focus Group), leading to greater self-efficacy. These reflections directly support quantitative findings that showed resilience mediating the positive impact of growth mindset and EI on academic buoyancy.

Finally, the role of social support emerged as particularly vital for managing academic stress and enhancing resilience, a point strongly emphasized in the focus group. Participants described support from peers, family, and instructors as crucial. One student highlighted peer support after a bad grade: “my friends were there for me… They reminded me that I wasn’t the only one… It made me feel supported and ready to try again” (Participant 2, Focus Group). Another recounted how an instructor’s belief in their potential provided “the confidence to keep going, even when things got tough” (Participant 14, Reflective Journal). Although not a quantitatively assessed pathway in our model, these findings suggest social support enhances resilience and buoyancy by normalizing struggles and fostering motivation, adding an important social dimension to the understanding of these individual psychological resources.

In summary, the qualitative findings offered nuanced insights into how students draw upon growth mindset, EI, and resilience to navigate their academic lives. Experiences of reframing setbacks, regulating emotions, persevering through challenges, and utilizing social support provided rich, practical examples of the mechanisms suggested by our quantitative model. These narratives underscore that individual psychological resources, often amplified by a supportive social context, collectively contribute to academic buoyancy by fostering resilience. The emergence of social support as a key facilitator highlights the interplay between individual capacities and the broader academic environment in promoting students’ ability to thrive.

## 5 Discussion

This study explored the intricate relationships between growth mindset, trait EI, resilience, and academic buoyancy among Chinese university students. The sequential explanatory mixed-methods design, integrating quantitative testing of a mediational model with qualitative insights into lived experiences, provided a comprehensive understanding of how these constructs contribute to academic success. The findings robustly indicate that both growth mindset and trait EI are positive predictors of resilience, which, in turn, exerts a significant and moderately strong positive influence on academic buoyancy. Notably, while multi-group analysis did not reveal statistically significant gender differences (*p* = 0.11), the qualitative data provided rich context, illustrating how students practically utilize these psychological resources to navigate daily academic challenges. The following discussion situates these results within existing literature and theoretical frameworks, addressing our key research hypotheses.

### 5.1 The role of growth mindset in predicting resilience and academic buoyancy

A core finding of this study centers on the significant predictive power of students’ growth mindset on both their resilience (H1) and, subsequently, their academic buoyancy (H2). This aligns with well-established evidence suggesting that a belief in the malleability of intelligence cultivates persistence when facing difficulties (Dweck, 2006; Yeager and Dweck, 2020; Claro and Loeb, 2024). From a social-cognitive standpoint (Bandura, 1986, 1997), the perception of ability as developable strengthens self-efficacy, thereby motivating students to re-engage with challenging material rather than disengaging. Indeed, the quantitative data demonstrated that students who endorsed growth mindset beliefs were more likely to report higher resilience, reflecting their capacity to “bounce back” from setbacks. From a theoretical perspective, this empirical link between growth mindset and general resilience—not just domain-specific persistence—within China’s demanding higher education system extends Dweck’s (2006) original framework. Such an extension underscores the adaptive utility of a growth mindset in a high-pressure cultural setting, suggesting robust cross-cultural applicability of mindset theory in fostering fundamental adaptive capacities (Li, 2017).

Furthermore, growth mindset not only predicted resilience but also showed a direct positive contribution to academic buoyancy, the ability to recover from routine academic stressors (Martin and Marsh, 2008). These pathways are consistent with previous research indicating that growth mindset interventions can enhance everyday academic coping skills (Macnamara and Burgoyne, 2023; Zhao et al., 2024). The qualitative findings further supported this, with students describing how they reframed academic obstacles as learning opportunities, consistent with Dweck’s (2006) concept of developable intelligence and recent evidence on mindset profiles showing adaptive goal orientations (Altikulaç et al., 2024). Student narratives emphasizing “trying again” and “learning from errors” exemplify this mindset’s role in fostering resolute responses to adversity, reinforcing assertions that growth mindsets are vital for academic persistence (Kim et al., 2022; Li and Bates, 2020). It is important to acknowledge that while contextual factors like socioeconomic status (King and Trinidad, 2021) or parental support (Chen et al., 2025) can moderate the impact of growth mindset, our findings highlight the broad applicability of growth mindset in enhancing resilience and academic buoyancy within the Chinese higher education context. This study therefore enriches social-cognitive theory by elucidating a specific cognitive pathway—belief in malleable intelligence—through which individuals’ self-systems can directly foster adaptive capacities like resilience and the more domain-specific academic buoyancy, offering tangible avenues for intervention aimed at bolstering student coping mechanisms.

### 5.2 The contribution of trait emotional intelligence to resilience and academic buoyancy

Similarly, trait EI emerged as another critical factor predicting students’ resilience (H3) and their academic buoyancy (H4), consistent with prior meta-analyses (Gkintoni et al., 2025; MacCann et al., 2020; Perera and DiGiacomo, 2013). This study confirmed that trait EI significantly predicts resilience, supporting existing research that emphasizes the role of emotional regulation in effective stress coping (Mayer and Salovey, 1997; Mohamed et al., 2025; Wong and Law, 2002). The qualitative data provided richer detail, with students describing how emotional regulation and self-awareness enabled them to overcome feelings of fear, anxiety, or hopelessness following academic setbacks. These accounts align with theories suggesting emotional competencies facilitate adaptive responses (Goleman, 2005). Moreover, trait EI demonstrated a significant direct effect on academic buoyancy. This suggests that emotionally intelligent students may proactively address routine setbacks by leveraging their emotional skills. Students skilled in perceiving and managing emotions (Wong and Law’s, 2002; SEA and ROE dimensions) tend to recover composure and re-engage with academic tasks more quickly. This emotional recovery process diminishes the likelihood of negative emotions, such as self-doubt or frustration, from becoming entrenched, thereby sustaining the motivation and effort necessary for academic buoyancy (Amponsah et al., 2024; Martin and Marsh, 2008). These data align with studies underscoring EI’s capacity to buffer against stress and test anxiety (Grover and Furnham, 2021; Mohamed et al., 2025; Trigueros et al., 2020). What these findings imply for models of trait EI (e.g., Wong and Law, 2002) is an extension beyond its established link to resilience; they provide robust empirical evidence for trait EI’s direct and indirect (via resilience) contributions to academic buoyancy. The qualitative data further refine this by highlighting how specific competencies like Self-Emotion Appraisal (SEA) and Regulation of Emotion (ROE) are practically applied in managing daily academic challenges, offering a nuanced understanding of which facets of trait EI might be particularly instrumental in fostering this day-to-day adaptiveness within the Chinese university context.

### 5.3 Resilience as a predictor of academic buoyancy and a key mediator

A key contribution of this research lies in demonstrating the pivotal role of resilience, not only as a direct predictor of academic buoyancy (H5) but also as a crucial mediator translating the benefits of both growth mindset (H6) and trait EI (H7) into enhanced academic buoyancy. While previous work has suggested resilience can buffer stress’s effects on academic outcomes (Bittmann, 2021; Masten, 2001; Nakhostin-Khayyat et al., 2024), our structural model provides robust empirical support for this specific mediational pathway. It indicates that resilience is not just an outcome, but an essential process variable that channels psychological resources like growth mindset and EI into effective coping with daily academic life. Students with a growth mindset and high EI are better positioned to respond adaptively to daily setbacks, and our findings show that resilience serves as the mechanism that effectively translates these positive orientations into consistent academic engagement and recovery (cf. Derakhshan and Fathi, 2025). The qualitative data echoed this, with participants frequently highlighting moments when setbacks felt overwhelming, yet resilience empowered them to reframe these experiences and persevere. This interplay between growth mindset, EI, and resilience emphasizes that academic buoyancy is not a fixed characteristic but a dynamically developed capability (Martin, 2013). Student reflections, such as viewing failure as “another chance to improve,” offer concrete examples of resilience in action. Consistent with Smith et al. (2008), participants noted that repeated experiences of “bouncing back” strengthened their confidence, making future setbacks less daunting, suggesting resilience builds over time, creating a positive cycle of coping and growth (García-Martínez et al., 2022; Montas et al., 2021). The empirical validation of this integrated mediational model, where resilience acts as a core transactive mechanism, offers a significant advancement to current understanding. For process models of resilience (e.g., Masten, 2001), which describe adaptive systems, this study specifies how particular cognitive (growth mindset) and emotional (trait EI) resources fuel these systems to produce a domain-specific adaptive outcome (academic buoyancy). Furthermore, this research enriches social-cognitive theory (Bandura, 1997) by demonstrating that resilience can be a key psychological pathway through which agentic personal factors influence adaptive functioning in response to everyday stressors. The result is a more granular, process-oriented understanding of how these psychological assets collectively operate, a perspective particularly valuable given that such an integrated model has not been extensively tested within the Chinese higher education context.

### 5.4 The emergent role of social support in fostering resilience and buoyancy

Beyond the hypothesized relationships, the qualitative phase also highlighted the significant role of social support in fostering resilience, an aspect not quantitatively measured in our model but vital for a holistic understanding. Peers, family, and instructors often played a crucial role in helping students regulate emotions, reframe challenges, and maintain a growth-oriented view. These findings align with research indicating that social support networks act as a scaffolding system that enhances resilience (Allan et al., 2014; Fu, 2024). Supportive professors or encouraging friends can buffer students from negative self-doubt, consistent with Collie et al.'s (2017) findings on the combined influence of social support and academic adversity on buoyancy. The prominence of social support in accounts from students selected for both high and low resilience suggests its importance regardless of baseline psychological attributes. Even students with strong growth mindsets or high EI emphasized the value of supportive relationships. Therefore, promoting social connectedness in universities may be another way to enhance resilience (Thomas and Allen, 2021), echoing ecological perspectives of resilience (Ungar, 2011; Ross et al., 2024) where supportive environments complement individual resources to promote thriving under stress. In terms of theoretical implications, the powerful emergence of social support from our qualitative data directly challenges the sufficiency of models that focus exclusively on intra-individual psychological resources to explain resilience and buoyancy. This finding strongly advocates for an extension of these individual-centric theories towards more comprehensive socio-ecological frameworks (Ross et al., 2024; Ungar, 2011). Our qualitative data imply that in collectivistic cultural contexts like China, the development, expression, and effectiveness of individual resources such as growth mindset and EI are likely deeply intertwined with, and potentially amplified by, the quality of social interactions and support systems, a crucial consideration for future theoretical development and intervention design. In conclusion, this study reinforces the significant contributions of growth mindset and trait emotional intelligence to student resilience and, subsequently, academic buoyancy. The qualitative data richly illustrate how students utilize these resources in the face of everyday academic stress and underscore the vital role of social support. Our findings advance social-cognitive theory and existing theories of mindset, emotional intelligence, and resilience by elucidating resilience’s key mediational role. They also demonstrate these constructs’ relevance within the Chinese higher education setting and highlight the necessity of integrating social-contextual factors into models of academic adaptation.

## 6 Implications and limitations

The findings of this study underscore significant practical implications for enhancing university curricula, student support services, and overall educational policy, particularly within the unique context of higher education in China. Given the positive predictive links from growth mindset and EI to resilience, and subsequently to academic buoyancy, integrating training in these psychological resources holds considerable promise.

This empowerment can begin with students in China, who, through a proactive understanding of these resources, can learn to consciously adopt a growth mindset when facing intense academic competition. Such an approach allows them to view demanding coursework or setbacks in high-stakes examinations as opportunities for strategic improvement rather than definitive failures. Similarly, developing trait EI skills, like emotional regulation and accurate self-appraisal, can help them manage stress from parental and societal expectations, maintain motivation during prolonged study (e.g., for postgraduate entrance exams), and build culturally valued supportive peer relationships.

Educators, in turn, play a critical role. Curriculum designers and faculty developers can incorporate targeted workshops and classroom activities to actively foster these resources. For instance, faculty can provide process-oriented feedback emphasizing effort, strategy, and learning progress over innate talent (Dweck, 2006; Yeager et al., 2019), thereby cultivating growth mindsets. They can also model emotional intelligence and create classroom climates where questioning and discussing challenges are encouraged, potentially reducing the “fear of losing face” common in collectivistic learning environments. Notably, such interventions need not be extensive; brief, structured tasks like group-based reflective journals (leveraging collectivistic strengths), mindset “priming” exercises, and peer-to-peer EI coaching can significantly bolster student resilience and buoyancy. This study’s mixed-methods evidence directly supports this, showing that students cultivating a growth mindset and using emotional regulation strategies exhibit greater capacity to recover from academic setbacks.

Although individual-level interventions are valuable, resilience, as highlighted qualitatively, is not solely an individual attribute; supportive environments are vital for students’ ability to bounce back. Therefore, university administrators and student affairs professionals in China should collaborate to cultivate a campus culture that normalizes mistakes as learning opportunities—a crucial shift in often performance-driven systems. They should actively encourage help-seeking, perhaps by destigmatizing and improving access to culturally attuned mental health support, while acknowledging the emotional demands of rigorous academic work in Chinese higher education. Specific strategies could include formal mentorship programs pairing senior with junior students, student-led study circles emphasizing collaborative learning over pure competition, and instructor feedback consistently emphasizing the developable nature of abilities (Yeager et al., 2019). Integrating these practices into university structures ensures that resilience development is not solely reliant on individual initiative, thereby more comprehensively fostering academic buoyancy.

Beyond institutional practices, this research also informs broader educational policy and future research in China. Theoretically, it reinforces the alignment of growth mindset, EI, and resilience with social-cognitive (Bandura, 1986, 1997) and positive psychology frameworks (Masten, 2018; Seligman, 2011), as results confirm that students’ self-beliefs and emotional self-regulation critically determine daily coping. Practitioners and researchers can use these insights to refine evidence-based interventions, especially in culturally specific contexts like Mainland China where academic pressure is high (Li, 2017). Future research, for example, could explore interventions integrating traditional Chinese values like perseverance with growth mindset principles. Furthermore, incorporating these constructs into teacher training is essential. This would equip pre- and in-service teachers to promote growth mindsets and emotional regulation, ultimately enhancing student resilience and academic buoyancy across the Chinese education system.

Given the intensely competitive academic landscape and strong emphasis on examination performance and career goals among Chinese university students (Zhao et al., 2024), this study’s focus on everyday academic buoyancy is particularly relevant for policymakers. University and governmental policymakers can use these findings to develop localized and systemic interventions. These might include reviewing and reforming academic evaluation systems that unduly penalize errors or foster unhealthy competition, which can undermine academic buoyancy. Policies could also promote integrating mental health literacy and psychological skill development (like EI and resilience) into core curricula, rather than as peripheral services. For instance, national guidelines encouraging universities to adopt a more holistic view of student success—valuing adaptive coping and well-being alongside grades—would be a significant step. Culturally adapted strategies, such as group-based reflective journals or EI training modules focusing on relational harmony and managing emotions tied to family/peer expectations, could effectively harness community-oriented values to strengthen resilience.

Despite its insights, this study has several limitations. First, while stratified random sampling was used quantitatively, findings may not fully generalize to all university students across China’s diverse higher education system; the small, purposefully selected qualitative sample (*N* = 20) offers depth rather than broad generalizability. Second, quantitative data relied on self-report measures, which, despite favorable common method variance checks, can be susceptible to social desirability or self-perception inaccuracies. Third, the cross-sectional quantitative design limits definitive causal inferences about the relationships between growth mindset, EI, resilience, and academic buoyancy; longitudinal studies are needed to explore these dynamics over time. Finally, the focus on Chinese university students, while providing valuable contextual insights, means the direct applicability of findings and the specific mediational model to other cultural contexts requires further comparative research.

## 7 Conclusion

This mixed-methods study investigated how growth mindset, trait EI, and resilience contribute to academic buoyancy among Chinese university students, yielding several key conclusions. Key findings indicate that a growth mindset—the belief in malleable abilities—and higher trait EI are significant psychological resources fostering student resilience. This cultivated resilience then directly enhances academic buoyancy, enabling students to effectively manage and recover from common academic stressors. Crucially, this research demonstrates resilience acts as a vital mediating mechanism, whereby enhanced resilience translates the benefits of growth mindset and trait EI into the practical capacity for academic buoyancy. Qualitative insights further refine this understanding, showing that while these individual psychological attributes are powerful, a supportive environment—particularly social support from peers, family, and faculty—often amplifies their effectiveness in promoting buoyancy. Ultimately, this study confirms academic buoyancy is not fixed but a dynamic, developable capacity. Higher education institutions can therefore enhance student success and well-being by implementing strategies that foster growth mindsets and EI competencies, primarily through building resilience within nurturing, supportive academic communities.

## Data Availability

The data analyzed in this study is subject to the following licenses/restrictions: the datasets generated and/or analyzed during the current study are available from the corresponding author, Ran Liu, on reasonable request. Requests should be directed to Lran1996@163.com and will be reviewed to ensure data sharing complies with ethical guidelines and participant confidentiality. Specifically, anonymized data, excluding any directly identifiable information, will be shared. Requests to access these datasets should be directed to Ran Liu, Lran1996@163.com.
